# Systematic analysis of expression profiles and prognostic significance for MMDS-related iron–sulfur proteins in renal clear cell carcinoma

**DOI:** 10.1038/s41598-022-22479-4

**Published:** 2022-11-16

**Authors:** Ling Yang, Yu-Xin Chen, Ying-Ying Li, Xiao-Juan Liu, Yong-Mei Jiang, Jia Mai

**Affiliations:** 1 Department of Laboratory Medicine, West China Second University Hospital, Sichuan University, Chengdu, China; 2 Key Laboratory of Birth Defects and Related Diseases of Women and Children (Sichuan University), Ministry of Education, Chengdu, Sichuan China

**Keywords:** Cancer, Cell biology, Oncology, Risk factors

## Abstract

Mitochondrial metabolism disorders play an important role in the occurrence and development of tumors, and iron–sulfur protein is an important molecule for maintaining the normal function of mitochondria. However, the relationship between the expression, prognostic value, and immune infiltration of MMDS-related iron–sulfur protein genes in kidney renal clear cell carcinoma (KIRC) remains unclear. Based on online databases bioinformatics analysis was performed to evaluate the expression differences, survival impacts, immune infiltration, and prognostic significance of multiple mitochondrial dysfunction syndrome (MMDS)-related iron–sulfur protein genes in KIRC patients. For example, the protein–protein interaction (PPI) network was constructed using STRING and GEPIA database; Survival impacts were constructed by TCGA database; Immune infiltration was analyzed using TIMER database. There were significant differences in the mRNA expression levels of ISCA1, ISCA2, C1ORF69 and NFU1 in KIRC among different tumor grades and individual cancer stages. Furthermore, KIRC with high transcription levels of ISCA1, ISCA2, C1ORF69 and NFU1 (p < 0.01) was significantly associated with long overall survival (OS) and disease-free survival (DFS). In addition, overexpression of four genes, NFU1, ISCA1, ISCA2, and C1ORF69 in KIRC indicated a better prognosis. Further studies showed that immune cells had a significantly positive correlation with iron–sulfur protein family genes, including CD8+ T cells, CD4+ T cells and B cells. More importantly, the results of immunohistochemistry showed that the expression of NFU1, ISCA1, ISCA2 and C1ORF69 in normal tissues was higher than that in renal clear cell carcinoma tissues. In this study, we systematically analyzed the expression and prognostic value of iron–sulfur protein family genes in KIRC. More importantly, NFU1, ISCA1, ISCA2, and C1ORF69 are expected to become potential therapeutic targets for KIRC, as well as potential prognostic markers for improving the survival rate and prognostic accuracy of KIRC.

## Introduction

Kidney cancer ranks among the top ten cancers in the world, accounting for 2% of the total global cancer incidence, and its incidence is on the rise every year^1^. For many years, kidney cancer patients have had almost no treatment options other than surgery, and survival times rarely exceed 1 year^2^. There are multiple histological subtypes of renal cancer, each characterized by a unique molecular landscape^3^. The most common subtype, KIRC, is an adenocarcinoma derived from renal tubular epithelial cells. It is the most common pathological subtype of renal cancer, accounting for approximately 75% of renal cancers. The prognosis of patients with metastatic KIRC is poor, with < 10% of patients surviving 5 years after diagnosis^3^. However, in the past decade, efforts have been made to study the mechanisms of the development, progression, and metastasis of KIRC^1,4,5^. Even with all this progress, there are many unanswered questions and unexplored avenues of research.

Iron–sulfur clusters are ancient and important cofactors that play important roles ranging from gene regulation to mitochondrial respiration in cells^6,7^. In eukaryotes, iron–sulfur cluster assembly is very complex, and more than 95% of the iron–sulfur cluster assembly is completed in the mitochondria. The mitochondrial assembly ISC system is highly conserved from prokaryotic to eukaryotic organisms and follows the “USA” synthetic model theory. The scaffold Protein ISCU receives inorganic iron ions and inorganic sulfur^8^ from a cysteine desulfurase complex consisting of NFS1 and ISD11 and assembles them into the simplest [2Fe–2S] clusters. Subsequently, some of the [2Fe–2S] clusters are dissociated by the chaperone protein and directly delivered to ISCU to form the mature [2Fe–2S] protein, and the other portion of the [2Fe–2S] clusters is delivered to ISCA1 and ISCA2 proteins for the generation of [4Fe–4S].

Many studies have reported that dysfunction of the iron–sulfur cluster assembly process can lead to diseases, especially diseases related to iron metabolism and energy metabolism, such as Friedreich’s ataxia, hereditary myopathy with lactic acidosis and X-linked sideroblastic anemia^8^. As an essential iron–sulfur (Fe/S) protein, NFU1 is involved in a variety of metabolic pathways and energy production and serves as a maturation factor for respiratory complex II (SDH)^9^. At present, some studies have found that NFU1 mutation may be related to pheochromocytoma and leukoencephalopathy with cysts and hyperglycinemia^10,11^. Preliminary studies have shown that ISCA1, ISCA2, and C1ORF69 participate in the maturation of [4Fe–4S] clusters as cofactors and are essential for mitochondrial energy metabolism. However, their role in tumors is not clear. Mitochondria play a vital role in the occurrence and development of tumors^12^, which makes us curious about the role iron–sulfur proteins play in tumors. Unfortunately, current research in this field is still very scarce.

According to the available studies, multiple mitochondrial dysfunction syndrome (MMDS) is classified into 5 types, caused by defects in NFU1, BOLA3, ISCA1, ISCA2, and C1ORF69 genes respectively, so we have selected these 5 related genes for further study^13^. Based on multiple large databases, bioinformatics analysis was performed to study the expression difference, survival impact, immune infiltration, and prognostic significance of the MMDS-related iron–sulfur protein genes in KIRC patients and their relationship with KIRC staging and grading. In addition, we analyzed the predictive functions and pathways of the MMDS-related iron–sulfur protein genes and their 100 frequently changed genes. This may help to further understand their potential role in KIRC.

## Materials and methods

### Data acquisition and processing

mRNA expression datasets and corresponding clinical information of TCGA-KIRC were obtained from The Cancer Genome Altas (TCGA) dataset (https://portal.gdc.cancer.gov/), in which the method of acquisition and application complied with the guidelines and policies. Data were analyzed with the R (version 4.0.3) and R Bioconductor packages. These data are an open resource, and no ethical issues were involved.

### Protein interaction network (PPI) network construction and app analysis

The five MMDS-elated iron–sulfur protein genes indicated a distinct set of interactions and networks. The protein–protein interaction (PPI) network was constructed using STRING database (https://cn.string-db.org/). We used the “similar genes detection” module of GEPIA to obtain the top 100 co-expression genes of the four gene’s signature. The top 100 co-expression genes corresponding proteins’ protein–protein interaction (PPI) network was constructed using Cytoscape software. These genes were import Cytoscape using stringAPP plugin. The PPIs with combined scores greater than 0.4 were selected for constructing PPI networks.

### Functional and pathway enrichment analysis

Gene annotation (GO) and Kyoto encyclopedia of genes and genomes (KEGG) analyses were performed to determine the potential biological functions of the top 100 co-expression genes by using the clusterProfiler R package. GO and KEGG enrichment analysis was according to the threshold of p < 0.05 and q < 1.

### Survival prognosis analysis

We used the “Survival Map” module of GEPIA (http://gepia2.cancer-pku.cn/) to obtain the Overall survival (OS) and disease-free survival (RFS) significance map date of MMDS-related iron–sulfur protein genes across all TCGA tumors. Cutoff-high (50%) and cutoff-low (50%) values were used as the expression thresholds for splitting the high-expression and low-expression cohorts^14–16^. The prognostic value of the MMDS related iron–sulfur protein genes mRNA expression of KIRC was also evaluated using GEPIA. All data was analyzed by Student’s t test. A p value of < 0.05 was considered to indicate statistical significance.

### Immune infiltration analysis

Among these five MMDS-related genes, ISCA1, ISCA2, C1ORF69 and NFU1 were found to be significantly altered in KIRC in our previous study. These findings suggested that ISCA1, ISCA2, C1ORF69 and NFU1 may function as tumor suppressors in KIRC (Fig. 2). So, to investigate the specific roles played by these four genes in KIRC, we exploreed the association between ISCA1, ISCA2, C1ORF69 and NFU1 expression and immune infiltrates across TCGA-KIRC. We used the “Immune-Gene” module of the TIMER (http://timer.cistrome.org/) to explore the association between ISCA1, ISCA2, C1ORF69 and NFU1 expression and immune infiltrates across TCGA-KIRC. The immune cells of CD8+ T-cells, CD4+ T-cells, B cells, NK cells and macrophage cells were selected. The EPIC algorithms was applied for immune infiltration estimations. The estimated P value were calculated to evaluate the associations between MMDS-related iron–sulfur protein genes and immune infiltration cells.

### Construction of the risk assessment model

The logistic LASSO model is a shrinkage method that can actively select from a large and potentially multicollinear set of variables in the regression, resulting in a more relevant and interpretable set of predictors. With the use of least absolute shrinkage and selection operator (LASSO) regression analysis, we calculated the individualized risk score with coefficients to construct a prognostic signature separating the high-risk and low-risk groups.

Meanwhile, the KIRC patients were divided into high-risk and low-risk groups according to the median risk score value. And then the risk score KM survival analysis with log-rank test were also used to compare the survival difference between above two groups. TimeROC (v 0.4) analysis was performed to compare the predictive accuracy of each gene and risk score.

### Immunohistochemistry analysis

KIRC tissue chip (shanghai outdo biotech Co. Ltd, HKidE020PG01) were fixed in 4% paraformaldehyde (PFA), embedded in paraffin. Immunohistochemical staining of the paraffin-embedded tumour tissues was performed using ISCA1 (bs-15417R, bioss), ISCA2 (13200-1-AP, proteintech), C1orf69 (bs-15075R, bioss) and NFU1 (A7097, ABclonal) primary antibodies and an ABC Elite immunoperoxidase kit according to the manufacturer’s instructions. Subsequently, all visual fields were observed under an optical microscope, and the brown particles in the cell cytoplasm were stained positive. The immunohistochemistry analysis involving human participants of this study has been admitted by the Institutional Review Board of West China Second University Hospital (registry number 2: 021-069) in March. 2021. We confirm that all methods were performed in accordance with the relevant guidelines and regulations.

### CRISPR-Cas9-mediated gene disruption

The sgRNA was designed according to the CDS region of ISCA2 gene searched in NCBI (Table 1), and then, the prepared sgRNA was constructed on Lenti-crisprV2 vector. The RCC-7860 cells were infected after the virus was packaged, 48 h after transfection, puromycin was added to the culture medium to select transfected cells. And then, cells were plated into 96-well plates at a density of approximately 1 cell/well. Such limiting dilutions were carried out several times to ensure that ISCA2 gene knockout monoclonal populations were obtained. RCC-7860 cells were maintained in 1640 medium supplemented with 10% heat-inactivated fetal bovine serum (Gibco) at 37 °C, 5% CO2.

**Table 1:** The target sequences for sgRNA against the human ISCA2 gene.

Sg sequence	Sense (5′–3′)	Antisense (5′–3′)
ISCA2-sgRNA1	CACCGTCTCTGCGTCGCGGCCGTTA	AAACTAACGGCCGCGACGCAGAGAC
ISCA2-sgRNA2	CACCGCGCCTGGGGGTCGTCCCTAA	AAACTTAGGGACGACCCCCAGGCGC

### Cell migration and invasion assay

Assays were performed in 24-well Boyden chambers (FALCON, 353097) transwell inserts for invasion assays. Tumor cells were seeded inside transwell inserts containing 200 μl culture media without FBS. As a chemoattractant, 600 μl culture media containing 10% FBS was placed in the lower chamber. After 18–24 h, cells that translocated to the lower surface of filters were fixed in 4% formaldehyde, stained with 0.1% crystal violet solution, and counted using a light microscope.

### Establishment of the four-gene-based prognostic gene signature

Univariate and multivariate cox regression analysis was performed to identify the appropriate terms to construct the nomogram. The forest was used to show the P value, HR and 95% CI of each variable through “forestplot” R package. A nomogram was developed based on the results of multivariate Cox proportional hazards analysis to predict the 1-year, 2-year, 3-year and 5-year overall recurrence. The nomogram provided a graphical representation of the factors, which can be used to calculate the risk of recurrence for an individual patient by the points associated with each risk factor through “rms” R package.

### Statistical analysis

Statistical analyses were performed using R version 4.0.3. Survival curves were plotted by the Kaplan–Meier method. For Kaplan–Meier curves, p-values and hazard ration (HR) with 95% confidence interval (CI) were generated by log-rank tests and univariate Cox proportional hazards regression. All other comparisons were analyzed by unpaired two-tailed Student’s t test. A p value of < 0.05 was considered to indicate statistical significance.

### Ethics approval and consent to participate

The study has been admitted by the Institutional Review Board of West China Second University Hospital. All the datasets were retrieved from the online databases, so it was confirmed that all written informed consent had already been obtained.

## Results

### Correlation of the MMDS-related iron–sulfur protein gene

**Figure 1:**
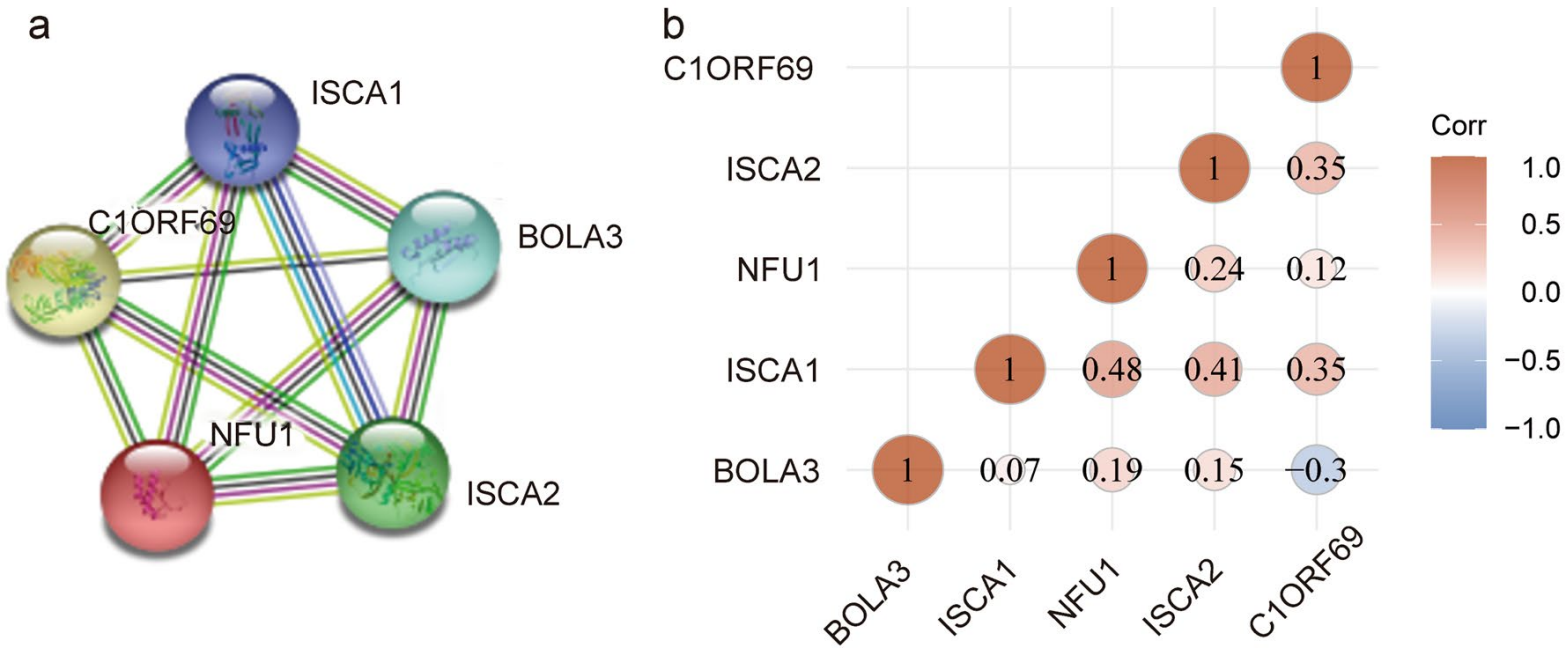
Correlation of the MMDS-related iron–sulfur protein gene and its expression with prognostic value in cancer. (**a**) PPI network. (**b**) Correlations of different iron–sulfur protein genes with each other in KIRC (Draw by the website tool: clinical bioinformatics (https://www.aclbi.com/static/index.html#/)).

The iron–sulfur protein family is composed of multiple iron–sulfur proteins, more than 200 different enzymes and proteins containing iron–sulphur clusters, known as iron–sulphur proteins, have been reported to have been identified to date^17,18^. Here, we selected five iron–sulfur proteins related to multiple mitochondrial dysfunction syndrome (MMDS) to study their relationship with cancer. We conducted a protein–protein interaction (PPI) network analysis of the MMDS-related iron–sulfur proteins with STRING to explore the potential interactions among them. STRING database analysis showed the following values: number of nodes = 5, number of edges = 10, average node degree = 4, and protein–protein interaction (PPI) enrichment p < 1.0e−16. These results indicated that there was an interaction among the MMDS-related iron–sulfur protein genes (Fig. 1a). Moreover, we also determined the correlations between MMDS-related iron–sulfur proteins by analyzing their mRNA expression by the R software package ggstatsplot for KIRC (The Cancer Genome Atlas Provisional, TCGA), and Pearson’s correction was included. The results indicated significant and positive correlations in the following iron–sulfur proteins: NFU1 with ISCA1; ISCA1 with ISCA2; and C1ORF69 with ISCA1 and ISCA2 (Fig. 1b). In addition, we also analyzed the expression of NFU1, ISCA1, ISCA2, C1ORF69 and BLOA3 genes in various tumor types through the oncomine database. The results showed that the expression of these four genes was significantly altered in KIRC.

### Prognostic features of the expression of distinct MMDS-related iron–sulfur proteins in KIRC

**Figure 2:**
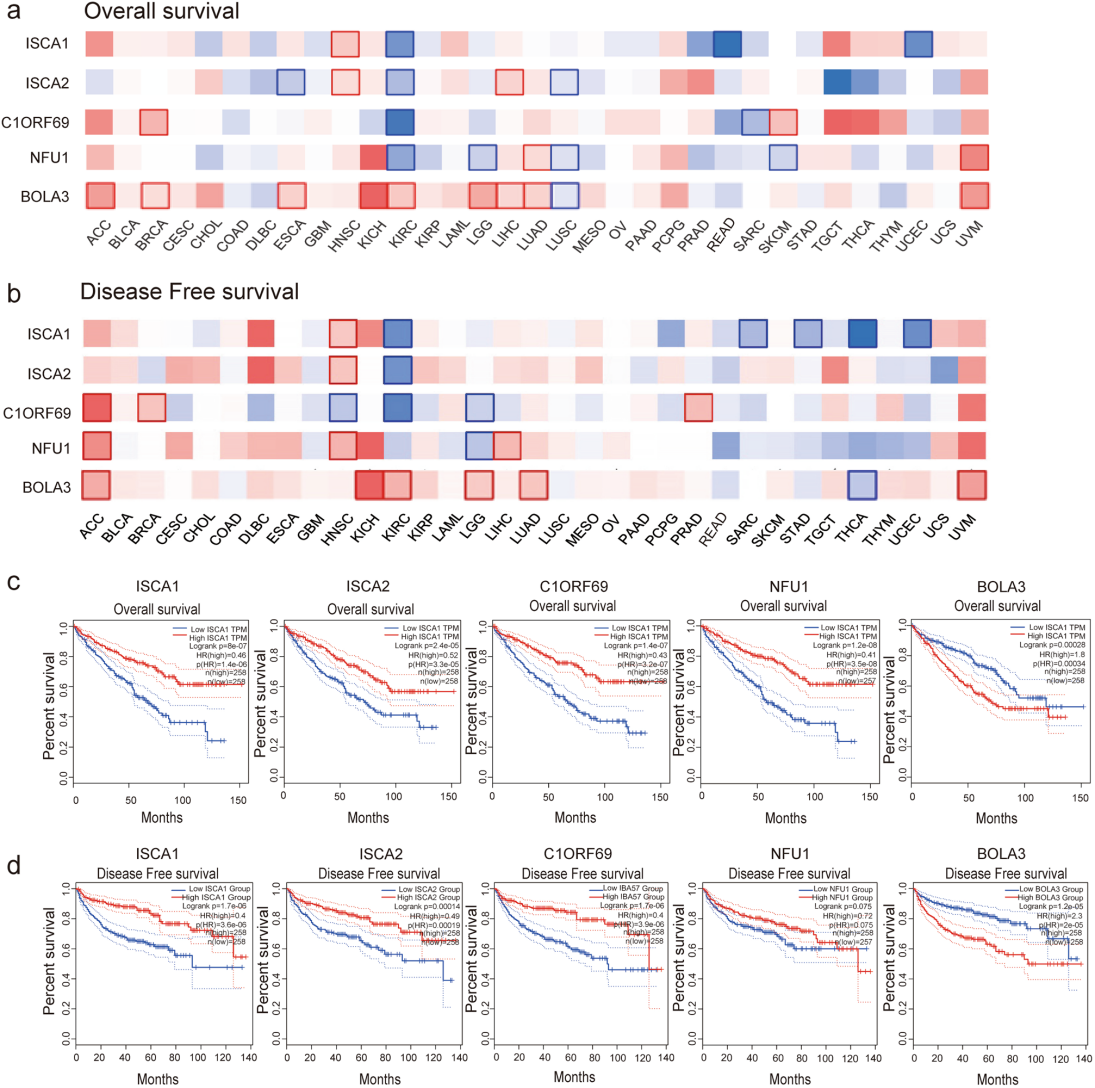
Prognostic feature of the mRNA expression among different MMDS related iron–sulfur protein genes in different types of cancers. Survival map of (**a**) overall survival (GEPIA), (**b**) and disease-free survival (GEPIA) of ISCA1, ISCA2, IBA57, NFU1 and BOLA3. (**c**) Kaplan–Meier curves for MMDS related iron–sulfur protein genes in KIRC patients (OS); (**d**) Kaplan–Meier curves for MMDS related iron–sulfur protein genes in KIRC patients (RFS). *ACC* adrenocortical cancer, *BLCA* bladder cancer, *BRCA* breast cancer, *CESC* cervical cancer, *CHOL* bile duct cancer, *COAD* colon cancer, *DLBC* large B-cell lymphoma, *ESCA* esophageal cancer, *GBM* glioblastoma, *HNSC* head and neck cancer, *KICH* kidney chromophobe, *KIRC* kidney clear cell carcinoma, *KIRP* kidney papillary cell carcinoma, *LAML* acute myeloid leukemia, *LGG* lower grade glioma, *LIHC* liver cancer, *LUAD* lung adenocarcinoma, *LUSC* lung squamous cell carcinoma, *MESO* mesothelioma, OV ovarian cancer, *PAAD* pancreatic cancer, *PCPG* pheochromocytoma & paraganglioma, *PRAD* prostate cancer, *READ* rectal cancer, *SARC* sarcom, *SKCM* melanoma, *STAD* stomach cancer, *TGCT* testicular cancer, *THCA* thyroid cancer, *THYM* thymoma, *UCEC* endometrioid cancer, *UCS* uterine carcinosarcoma, *UVM* ocular melanomas.

To evaluate the value of the differentially expressed MMDS-related iron–sulfur proteins in the progression of different tumors, GEPIA was used to analyze the correlation between NFU1, BOLA3, ISCA2, ISCA1, and C1ORF69 mRNA expression levels and clinical results. The analysis results of overall survival (OS) and disease-free survival (RFS) are presented in Fig. 2a,b. The results indicate that the differential expression of MMDS-related iron–sulfur proteins is significant in a variety of tumors. KIRC with high transcription levels of ISCA1, ISCA2, C1ORF69 and NFU1 was significantly associated with a longer OS (Fig. 2c, p < 0.01) and DFS (Fig. 2d, p < 0.01). Meanwhile, KIRC with high transcription levels of BOLA3 (p < 0.01) was significantly associated with a shorter OS (Fig. 2c, p < 0.01) and DFS (Fig. 2d, p < 0.01). These findings suggest that ISCA1, ISCA2, C1ORF69 and NFU1 may function as tumor suppressors in KIRC.

### The mRNA expression levels of MMDS-related iron–sulfur protein genes in KIRC of different pathological stages

**Figure 3:**
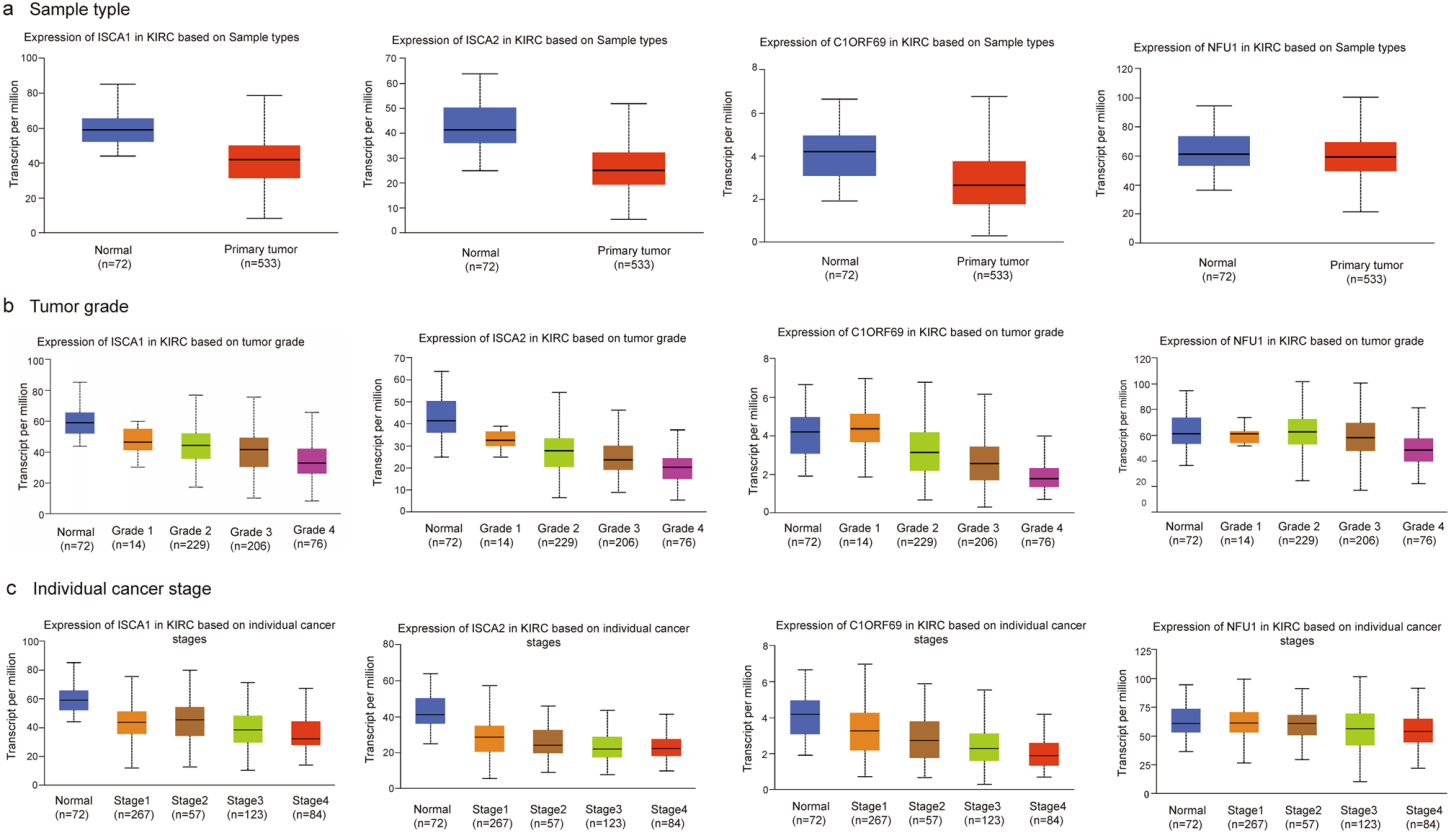
The mRNA expression levels of the MMDS related iron–sulfur protein genes in KIRC of different pathological stages. (**a**) Between tumor and normal tissues. (**b**) Among different tumor grades. (**c**) Among different individual cancer stages.

We evaluated the expression differences of ISCA1, ISCA2, C1ORF69 and NFU1 normal tissues versus tumor tissues and among tumor tissues of KIRC. The results showed that the expression levels of ISCA1, ISCA2, C1ORF69 and NFU1 were significantly different in normal tissues versus KIRC tumor tissues (Fig. 3a, p < 0.01), among different tumor grades (Fig. 3b, p < 0.01), and among different individual cancer stages (Fig. 3c, p < 0.01).

### Correlation between mRNA expression of MMDS-related iron–sulfur protein genes and tumor-infiltrating immune cells in KIRC patients

**Figure 4:**
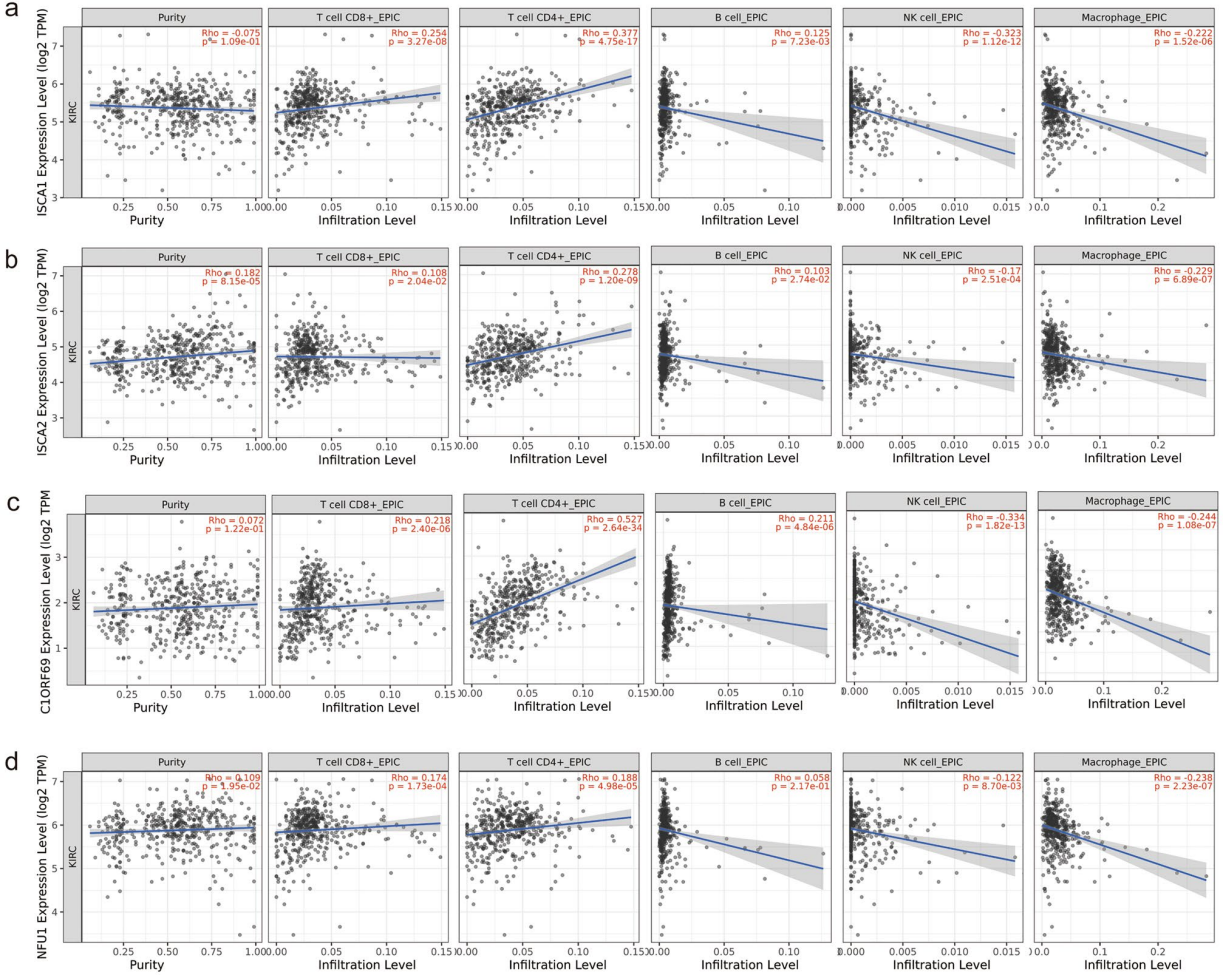
Correlation between the MMDS related iron–sulfur protein genes and tumor-infiltrating immune cells.

Infiltrating immune cells play an important role in the tumor microenvironment and are closely related to the occurrence, progression and metastasis of cancer^19^. Based on the TIMER database, we investigated the potential relationship between the infiltration level of different immune cells and MMDS-related iron–sulfur protein gene expression. We observed a statistically positive correlation between ISCA1 and C1ORF69 expression levels and the immune infiltration of CD8+ T cells in KIRC. Moreover, there was a statistically positive correlation between NFU1, ISCA1, ISCA2 and C1ORF69 expression levels and the immune infiltration of CD4+ T cells in KIRC. We also observed a statistically positive correlation between the immune infiltration of B cells and C1ORF69 expression levels in KIRC. The results showed a statistically negative correlation between NFU1, ISCA1, ISCA2 and C1ORF69 expression levels and immune infiltration of NK cells and macrophages (Fig. 4).

### Construction and validation of the 4-gene signature based on MMDS-related iron–sulfur protein genes

**Figure 5:**
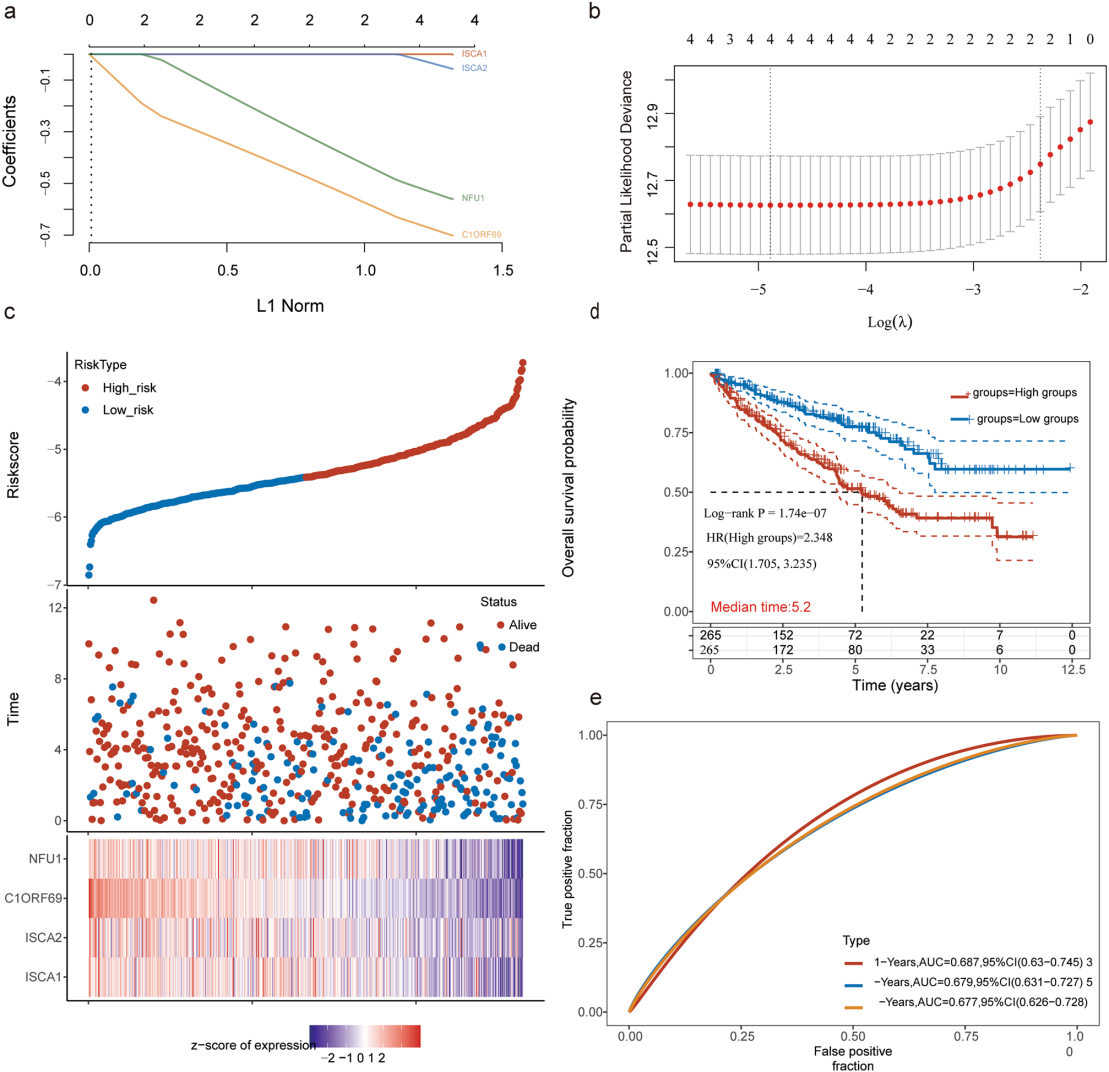
Construction and validation of the 4-gene signature based on the MMDS related iron–sulfur protein genes. (**a**) The changing trajectory of each independent variable. (**b**) The confidence interval under each lambda. (**c**) Construction of risk model and distributions. (**d**) The KM survival curve distribution of the 4-gene signature in all TCGA datasets. (**e**) The 1-year, 3-year and 5-year ROC of the nomogram.

We performed the LASSO regression method to find the most significantly prognostic genes. The change trajectory of independent variables showed that the number of independent variable coefficients tending toward zero gradually increased with the gradual decrease in lambda. The tenfold cross-validation method was used to build the model, and the CI under each lambda was analyzed. The results showed that the model was optimal when log (lambda) =  − 2; therefore, we chose the four genes as target genes when log (lambda) =  − 2 (Fig. 5a,b). The risk model of the two genes is as follows: risk score = (− 1e−04) × ISCA1 + (− 0.0446) × ISCA2 + (− 0.6871) × C1ORF69 + (− 0.546) × NFU1.

Meanwhile, the KIRC patients were divided into high-risk and low-risk groups according to the median risk score value. The patients with a high risk score suffered higher survival risk, as shown in Fig. 5c. Moreover, the levels of 4 differentially expressed genes in the two groups are shown by the heatmap in Fig. 5c. Survival analysis showed significant differences between the high-risk and low-risk groups (p < 0.01, Fig. 5d). Moreover, the area under the curve (AUC) values for the 1-year, 3-year and 5-year survival rates of the risk model in Fig. 5e were 0.687, 0.679, and 0.677, respectively, indicating the superior predictive accuracy of the risk model for the prognosis of KIRC.

**Figure 6:**
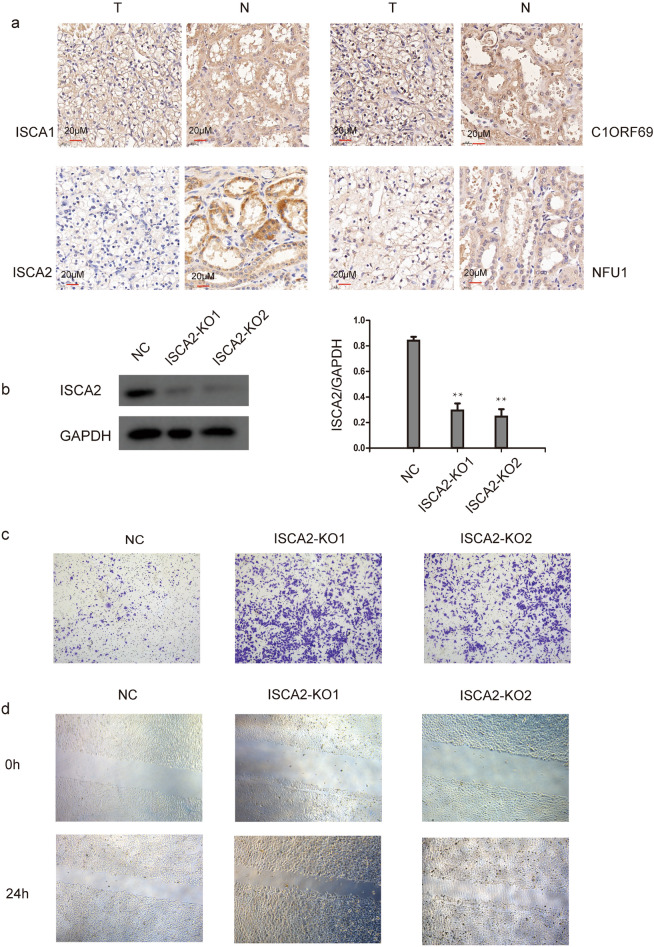
The expression of the MMDS related iron–sulfur protein genes in KIRC (IHC). (**a**) The expression of the MMDS related iron–sulfur protein genes in KIRC, N (normal kidney tissue), T (kidney tumor tissue); (**b**) WB experiment is used to verify the knockout effect (the blots cut prior to hybridization with antibodies). (**c**) The migratory ability of ISCA2 deficiency in RCC-7860 cells was assayed using an uncoated transwell assay. (**d**) Cell migration of RCC-7860 cells after ISCA2 deficiency was measured by wound healing assay (24 h).

In addition, we performed immunohistochemistry (IHC) to test MMDS-related iron–sulfur protein expression in KIRCtissues and their counterparts. We found that NFU1, ISCA1, ISCA2, and C1ORF69 were more highly expressed in normal tissues than in KIRCtissues (Fig. 6a). Since ISCA2 gene has the most obvious difference in immunohistochemical results, we further proved the role of ISCA2 in KIRC with cell experiments. Then, we used CRISPR–Cas9 technology to knock out the ISCA2 gene of RCC-7680 cells and observe its effect on cell migration and invasion. Western blot was used to verify the knockout effect (Fig. 6b). In addition, the colony-forming ability of RCC-7680 cells was increased following ISCA2 knockout, accompanied by an increase in cell migratory ability (Fig. 6c,d). All these results indicated that ISCA2 deficiency increased KIRC malignant behaviour in vitro.

### Predicted functions of the changes in MMDS-related iron–sulfur protein gene factors in patients with KIRC

**Figure 7:**
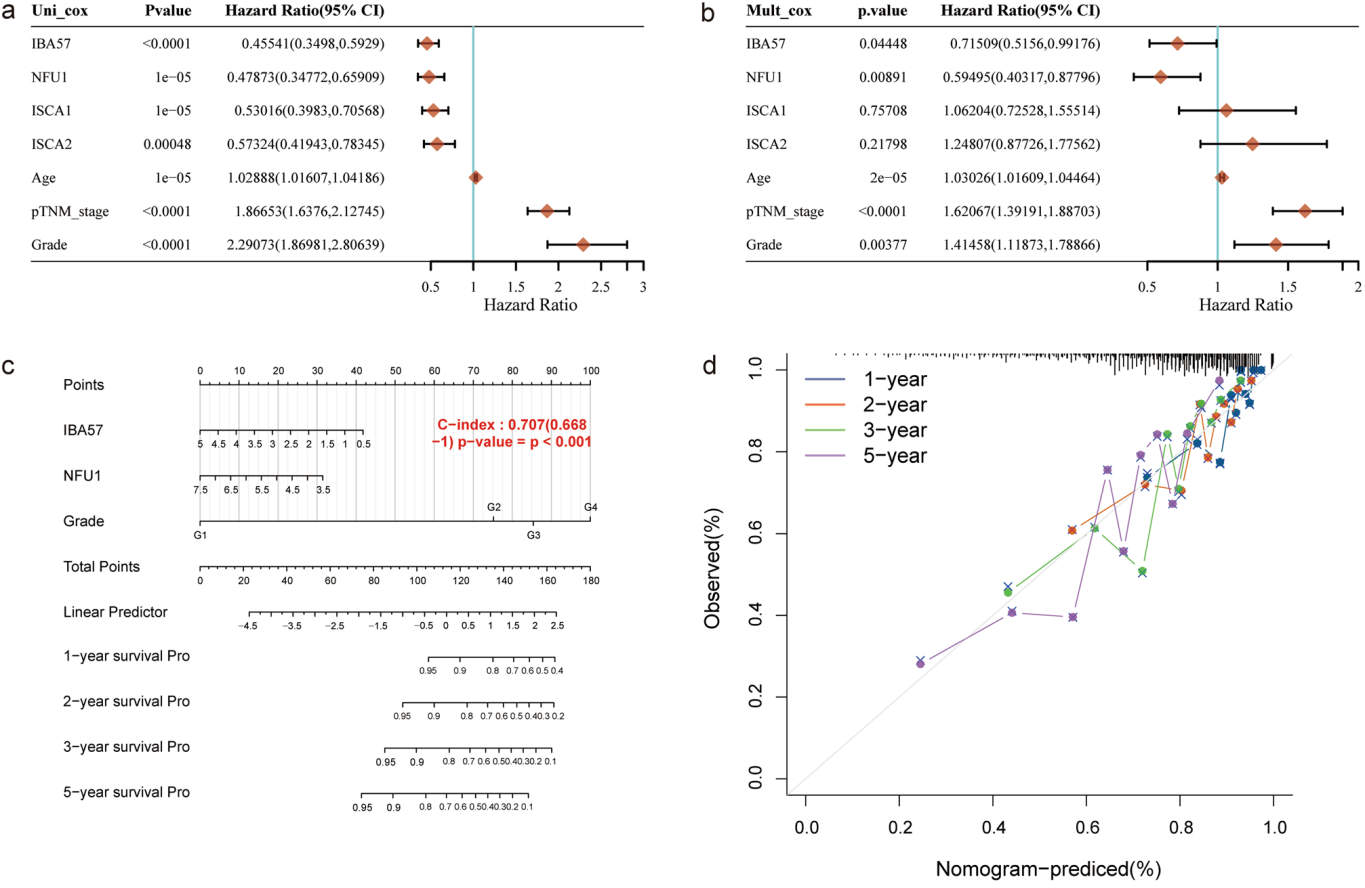
Construction and validation of the 4-gene signature based on the MMDS related iron–sulfur protein genes. (**a**) Univariate cox analysis of Iron–sulfur protein family genes in the TCGA-KIRC cohort. (**b**) Multivariate cox analysis of the MMDS related iron–sulfur protein genes in the TCGA-KIRC cohort. (**c**) Nomogram integrating risk score and clinical characteristics. (**d**) The 1-, 2-, 3- and 5-year calibration curves of the nomogram.

We sought to further explore the prognostic role of the MMDS-related iron–sulfur protein genes in KIRC. Four genes, NFU1, ISCA1, ISCA2, and C1ORF69, were identified by univariate Cox regression analysis in TCGA-KIRC datasets. The results showed that NFU1, ISCA1, ISCA2, and C1ORF69 were significantly related to the prognosis of KIRC. Forest maps can simply and intuitively show the statistical results of different factors, as shown in Fig. 7a: C1ORF69 (HR 0.455, p < 0.0001), NFU1 (HR 0.479, p = 0.0001), ISCA1 (HR 0.530, p = 0.0001), ISCA2 (HR 0.573, p = 0.00048) and grade (HR 2.291 p < 0.0001). After correction for other confounding factors, NFU1, ISCA1, ISCA2, and C1ORF69 were still proven to be independent predictors of OS in the multivariate Cox regression analysis (Fig. 7b C1ORF69 (HR 0.715, p = 0.044), NFU1 (HR 0.595, p = 0.009), and grade (HR 1.415, p = 0.004).

A nomogram was constructed with C1ORF69, NFU1, and grade. As observed from the results of the model, C1ORF69 and NFU1 have a great influence on survival outcome, indicating that the risk model based on C1ORF69 and NFU1 can predict the prognosis of KIRC (Fig. 7c). The calibration plot can be used to show the performance of the 1-year, 2-year, 3-year and 5-year nomograms (Fig. 7d). Furthermore, the nomogram performed well in predicting the prognosis of KIRC.

### GO and KEGG enrichment analysis of the 100 coexpressed genes of the 4 genes in KIRC patients

**Figure 8:**
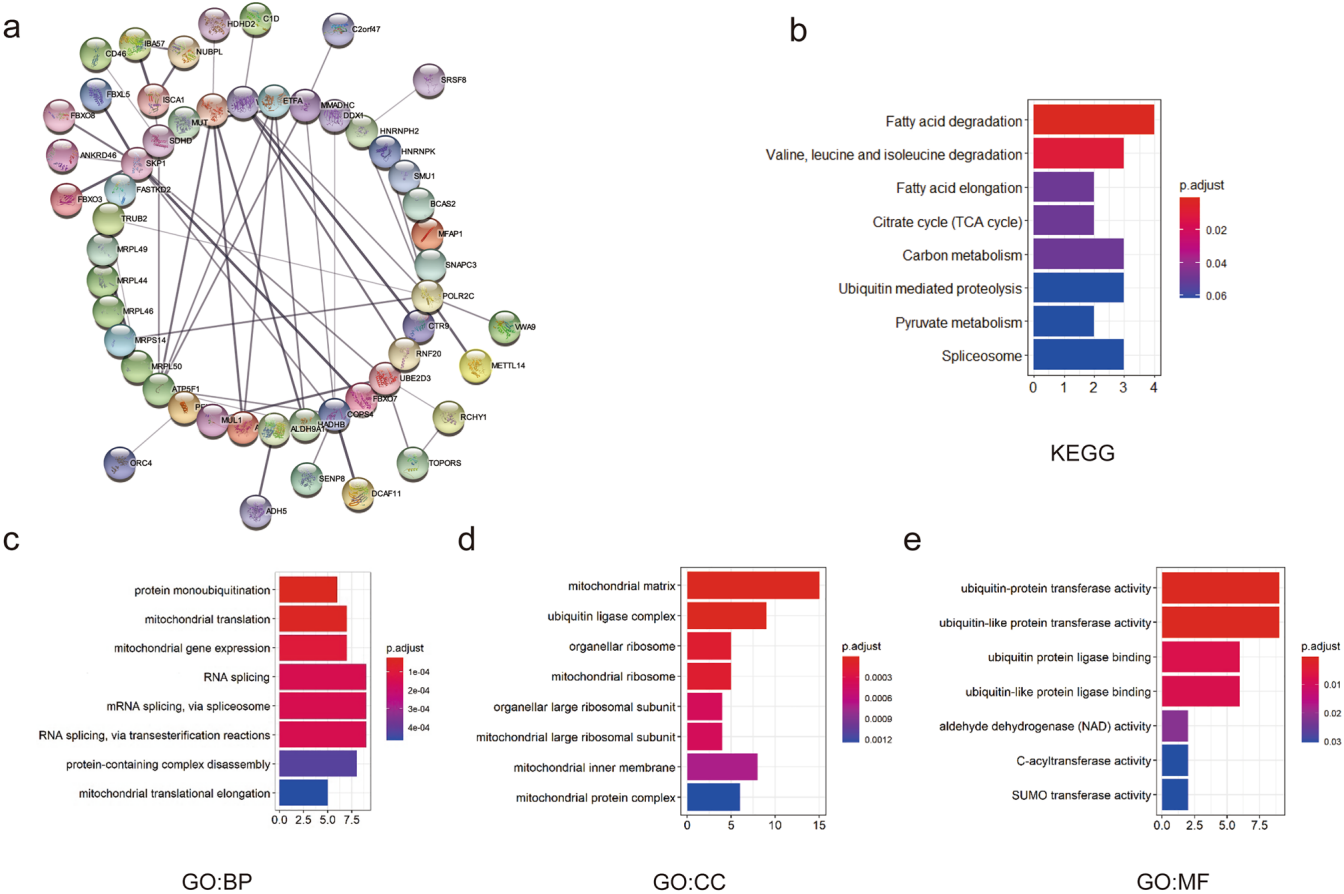
GO and KEGG enrichment analysis of the co-expression genes in KIRC patients. (**a**) The network of 100 most similar MMDS related iron–sulfur protein genes. The functions of the 100 most similar genes by (**b**). KEGG enrichment analysis, (**c**) GO biological processes, (**d**) GO cellular components, and (**e**) GO molecular functions.

After analyzing the prognostic value of NFU1, ISCA1, ISCA2, and C1ORF69 in KIRC patients, we analyzed 100 coexpressed genes that were found to be significantly associated with NFU1, ISCA1, ISCA2, and C1ORF69 by similar gene detection using GEPIA and listed them in Supplementary Table 1. We then constructed a network for the 100 most frequently altered neighboring genes of NFU1, ISCA1, ISCA2, and C1ORF69 using STRING. The results showed that the frequently altered neighboring genes SKP1, ETFA, FBXO3, FBXO7, FBXO8, ATP5F1 and POLR2C were closely associated with alterations in NFU1, ISCA1, ISCA2, and C1ORF69 (Fig. 8a).

To explore the pathways related to the functions of the frequently altered neighboring genes, we performed KEGG analysis^20^. As shown in Fig. 8b, the fatty acid degradation and elongation pathway, citrate cycle pathway, and carbon metabolism pathway were involved in the alterations of NFU1, ISCA1, ISCA2, and C1ORF69 in KIRC (Fig. 8b).

We used GO enrichment analysis to predict the functional role of target host genes from three aspects: biological processes (BP), cell components (CC), and molecular functions(MF). The results showed that BP, such as protein monoubiquitination, mitochondrial translation and mitochondrial gene expression were remarkably regulated by gene alterations in NFU1, ISCA1, ISCA2, and C1ORF69 in KIRC. In addition, CC, including the mitochondrial matrix, ubiquitin ligase, mitochondrial ribosomes and mitochondrial protein complexes were also significantly controlled by alterations in those 4 genes, and they also prominently affected MF, such as ubiquitin-protein transferase activity, ubiquitin-like protein transferase activity and aldehyde dehydrogenase (NAD) activity (Fig. 8c–e).

## Discussion

Mitochondria are organelles involved in many cellular functions. In addition to their central role in metabolism, they are also involved in biological processes, such as cell death, ROS production and immune responses^21,22^. Studies have shown that functional changes in mitochondria are critical to the adaptation and survival of cancer cells^20,23^. As an important part of the respiratory chain complex, iron–sulfur clusters play an important role in maintaining the normal function of mitochondria. However, the role of the iron–sulfur protein family in tumors is not clear. The iron–sulfur protein family is composed of multiple iron–sulfur proteins. Here, we selected five iron–sulfur proteins related to multiple mitochondrial dysfunction syndrome (MMDS) to study their relationship with cancer.

In our research, we found that NFU1, ISCA1, ISCA2, C1ORF69, and BLOA3 were altered significantly in kidney cancer tissues in multiple datasets. Moreover, there were significant differences in the mRNA expression levels of ISCA1, ISCA2, C1ORF69 and NFU1 in KIRC among different tumor grades and individual cancer stages. Furthermore, the results indicated that KIRC with high transcription levels of ISCA1, ISCA2, C1ORF69 and NFU1was significantly associated with long OS and DFS. These results give us more confidence that the iron–sulfur protein family may play a tumor suppressor role in the occurrence and development of renal cell clear cell carcinoma.

Furthermore, we tried to explore the prognostic role of iron–sulfur protein family genes in KIRC. The results showed that some iron–sulfur protein family genes can be used as prognostic indicators for patients. For example, overexpression of four genes, NFU1, ISCA1, ISCA2, and C1ORF69, in KIRC indicated a better prognosis. In addition, the multivariate analysis results indicated that the signatures were not only independent predictors of KIRC in the clinic but also potential therapeutic targets for cancer patients in the future.

In addition, we found a significant correlation of these signatures to immune cells according to the TIMER datasets. Further studies showed that immune cells had a significantly positive correlation with iron–sulfur protein family genes, including CD8+ T cells, CD4+ T cells and B cells. Therefore, iron–sulfur protein genes could promote humoral and cellular immune processes and enhance immune surveillance and immune response, thereby inhibiting the development of cancer. Nevertheless, further research is needed to confirm the mechanism of these features in the immune process.

The results of the present study showed that mitochondrial energy metabolism was remarkably regulated by iron–sulfur protein family alterations in KIRC. Under normoxic and hypoxic conditions, a high rate of glycolysis can promote the growth of cancer. The glycolysis process can produce ATP to support proliferation and acidify the local tumor environment, which helps cancer cells escape the recognition and metastasis of immune cells^12,21,24,25^. Based on previous studies, we suspect that changes in iron–sulfur protein family genes affect mitochondrial translation and mitochondrial gene expression, thereby preventing the transformation of cancer cells from aerobic respiration to glycolysis and limiting the development of tumors in terms of energy metabolism. The results of immunohistochemistry showed that the expression levels of NFU1, ISCA1, ISCA2 and C1ORF69 in normal tissues were higher than those in renal clear cell carcinoma tissues, which further verified our previous hypotheses. More convincingly, in cell experiments, knocking out the ISCA2 gene of RCC-7860 cells enhanced cell migration and invasion capabilities, which further confirmed our hypothesis. Despite these findings, almost no study had been conducted on KIRC thus far. Further research is needed to elucidate the specific mechanism of iron–sulfur protein family genes in the development of KIRC.

Taken together, we systematically analyzed the expression and prognostic value of iron–sulfur protein family genes in KIRC to obtain a deep understanding of the complexity of the molecular biology of KIRC. Our results indicate that increased expression of NFU1, ISCA1, ISCA2, and C1ORF69 may play an important role in KIRC tumorigenesis. More importantly, NFU1, ISCA1, ISCA2, and C1ORF69 are expected to become potential therapeutic targets for KIRC, as well as potential prognostic markers for improving the survival rate and prognostic accuracy of KIRC.

## Supplementary Information

Supplementary Information.

Supplementary Table 1.

## Data Availability

The current research and analysis data sets come from online databases, including: the Cancer Genome Atlas (TCGA, http://can-cergenome.nih.gov/), Gene Expression Omnibus (GEO, https://www.ncbi.nlm.nih.gov/geo/), and TIMER (http://timer.cistrome.org/). Other data supporting the results of this study can be obtained from the corresponding author upon reasonable request.

## Abbreviations

KIRC: Kidney renal clear cell carcinoma
MMDS: Multiple mitochondrial dysfunctions syndrome
OS: Overall survival
DFS: Disease-free survival
TCGA: The cancer genome altas
PPI: The protein–protein interaction
GO: Gene annotation
KEGG: Kyoto encyclopedia of genes and genomes
LASSO: The least absolute shrinkage and selection operator
IHC: Immunohistochemistry

## References

[1] Ma X, Wang X, Dong Q, Pang H, Xu J, Shen J, Zhu J (2021). Inhibition of KIF20A by transcription factor IRF6 affects the progression of renal clear cell carcinoma. Cancer Cell Int..

[2] Owens B (2016). Kidney cancer. Nature.

[3] Turajlic S, Swanton C, Boshoff C (2018). Kidney cancer: The next decade. J. Exp. Med..

[4] Martínez-Sáez O, Gajate Borau P, Alonso-Gordoa T, Molina-Cerrillo J, Grande E (2017). Targeting HIF-2 α in clear cell renal cell carcinoma: A promising therapeutic strategy. Crit. Rev. Oncol. Hematol..

[5] Linehan WM, Ricketts CJ (2019). The cancer genome atlas of renal cell carcinoma: Findings and clinical implications. Nat. Rev. Urol..

[6] Lill R (2009). Function and biogenesis of iron–sulphur proteins. Nature.

[7] Beilschmidt LK, Puccio HM (2014). Mammalian Fe-S cluster biogenesis and its implication in disease. Biochimie.

[8] Sheftel A, Stehling O, Lill R (2010). Iron–sulfur proteins in health and disease. Trends Endocrinol. Metab..

[9] Wesley NA, Wachnowsky C, Fidai I, Cowan JA (2017). Understanding the molecular basis for multiple mitochondrial dysfunctions syndrome 1 (MMDS1): Impact of a disease-causing Gly189Arg substitution on NFU1. FEBS J..

[10] Seo SH, Kim JH, Kim MJ, Cho SI, Kim SJ, Kang H, Shin CS, Park SS, Lee KE, Seong MW (2020). Whole exome sequencing identifies novel genetic alterations in patients with pheochromocytoma/paraganglioma. Endocrinol. Metab. (Seoul).

[11] Nizon M, Boutron A, Boddaert N, Slama A, Delpech H, Sardet C, Brassier A, Habarou F, Delahodde A, Correia I (2014). Leukoencephalopathy with cysts and hyperglycinemia may result from NFU1 deficiency. Mitochondrion.

[12] Badrinath N, Yoo SY (2018). Mitochondria in cancer: In the aspects of tumorigenesis and targeted therapy. Carcinogenesis.

[13] Lebigot E, Schiff M, Golinelli-Cohen MP (2021). A review of multiple mitochondrial dysfunction syndromes, syndromes associated with defective Fe-S protein maturation. Biomedicines.

[14] Sun CC, Li SJ, Hu W, Zhang J, Zhou Q, Liu C, Li LL, Songyang YY, Zhang F, Chen ZL (2022). Retraction notice to: Comprehensive analysis of the expression and prognosis for E2Fs in human breast cancer. Mol. Ther..

[15] Cui X, Zhang X, Liu M, Zhao C, Zhang N, Ren Y, Su C, Zhang W, Sun X, He J (2020). A pan-cancer analysis of the oncogenic role of staphylococcal nuclease domain-containing protein 1 (SND1) in human tumors. Genomics.

[16] Hu Y, Zheng M, Zhang D, Gou R, Liu O, Wang S, Lin B (2021). Identification of the prognostic value of a 2-gene signature of the WNT gene family in UCEC using bioinformatics and real-world data. Cancer Cell Int..

[17] Bandyopadhyay S, Chandramouli K, Johnson Michael K (2008). Iron–sulfur cluster biosynthesis. Biochem. Soc. Trans..

[18] Saha PP, Vishwanathan V, Bankapalli K, D'Silva P (2018). Iron–sulfur protein assembly in human cells. Rev. Physiol. Biochem. Pharmacol..

[19] Steven A, Seliger B (2018). The role of immune escape and immune cell infiltration in breast cancer. Breast Care (Basel).

[20] Huang Y, Zhang G, Zhao R, Zhang D (2020). Aggregation-induced emission luminogens for mitochondria-targeted cancer therapy. ChemMedChem.

[21] Kafkova A, Trnka J (2020). Mitochondria-targeted compounds in the treatment of cancer. Neoplasma.

[22] Anderson RG, Ghiraldeli LP, Pardee TS (2018). Mitochondria in cancer metabolism, an organelle whose time has come?. Biochim. Biophys. Acta Rev. Cancer.

[23] Klein K, He K, Younes AI, Barsoumian HB, Chen D, Ozgen T, Mosaffa S, Patel RR, Gu M, Novaes J (2020). Role of mitochondria in cancer immune evasion and potential therapeutic approaches. Front. Immunol..

[24] Zong WX, Rabinowitz JD, White E (2016). Mitochondria and cancer. Mol. Cell.

[25] Yang J, Ren B, Yang G, Wang H, Chen G, You L, Zhang T, Zhao Y (2020). The enhancement of glycolysis regulates pancreatic cancer metastasis. Cell Mol. Life Sci..

