# Supplementary material for: Systematic analysis of expression profiles and prognostic significance for MMDS-related iron–sulfur proteins in renal clear cell carcinoma
Source: Sci Rep. 2022 Nov 16;12:19637. doi: 10.1038/s41598-022-22479-4 (PMC9669015; doi:10.1038/s41598-022-22479-4)
Supplement: Supplementary file 1 — Supplementary Information. [file 41598_2022_22479_MOESM1_ESM.docx]

**Supplemental Information file**

Systematic Analysis of Expression Profiles and Prognostic Significance for MMDS- related iron-sulfur proteins in Renal Clear Cell Carcinoma

Ling Yang^1 a^, Yu-Xin Chen^1 a^, Ying-Ying Li^1^, Xiao-Juan Liu^1^, Yong-Mei Jiang^1*^, Jia Mai^1*^

1 Department of Laboratory Medicine, West China Second University Hospital, Sichuan University;

2 Key laboratory of birth defects and related diseases of women and children (Sichuan University), Ministry of Education, Chengdu, Sichuan, China;

a Ling Yang and Yu-Xin Chen are co-first authors and contributed equally to this work

Correspondence: [jiangyongmeiwst@163.com](mailto:jiangyongmeiwst@163.com); [maijia@mail2.sysu.edu.cn](mailto:maijia@mail2.sysu.edu.cn)

**Supplemental Figure 1**


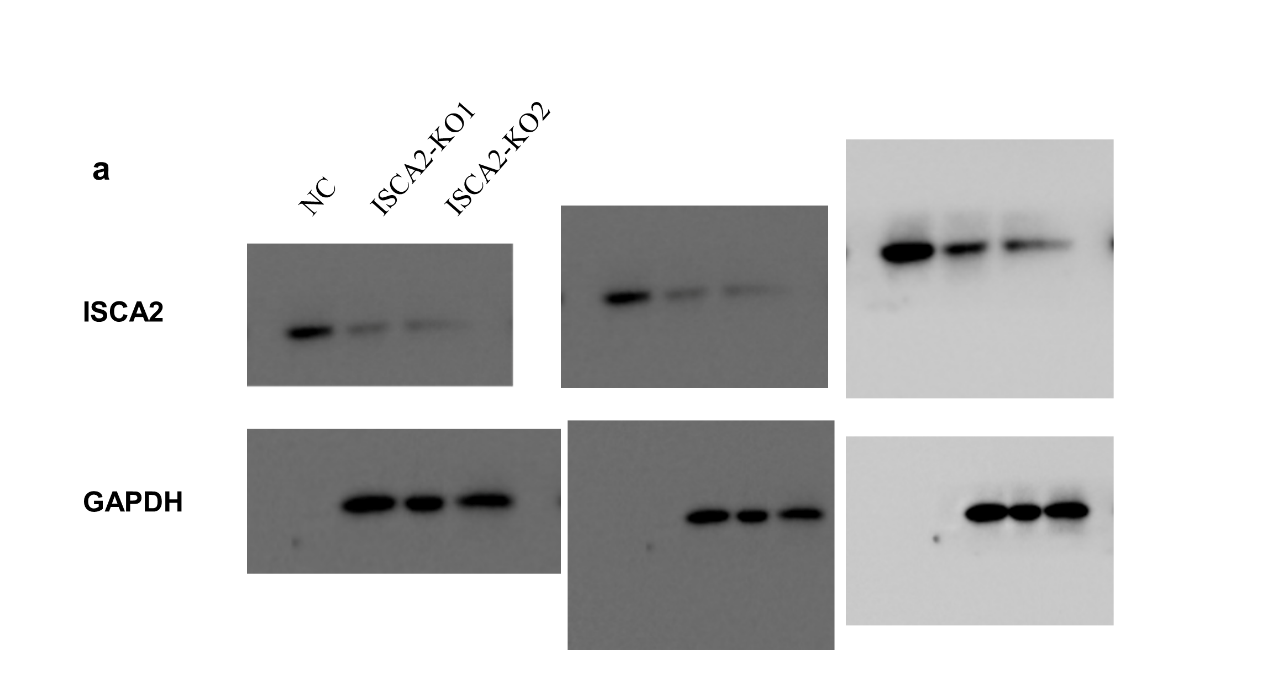


(a) WB experiment is used to verify the knockout effect;

**Supplemental Table**

**Supplemental Table 1** 100 Coexpressed Genes of the 4 Genes in KIRC Patients.
